# First person – Brandon Scharpf and Hannah Ruetten

**DOI:** 10.1242/dmm.052276

**Published:** 2025-01-24

## Abstract

First Person is a series of interviews with the first authors of a selection of papers published in Disease Models & Mechanisms, helping researchers promote themselves alongside their papers. Brandon Scharpf and Hannah Ruetten are co-first authors on ‘ Prostatic *Escherichia coli* infection drives CCR2-dependent recruitment of fibrocytes and collagen production’, published in DMM. Brandon is a PhD student in the lab of Dr Chad Vezina at University of Wisconsin-Madison, Madison, WI, USA, investigating the mechanisms of fibrosis-induced lower urinary tract dysfunction, which is necessary for the development of, currently unavailable, targeted treatments. Hannah is a postdoctoral research fellow in the lab of Dr Koudy Williams and Dr Steve Walker at Wake Forest Institute for Regenerative Medicine, Winston Salem, NC, USA, optimising animal models for understanding pathophysiology and developing treatments for genitourinary diseases.


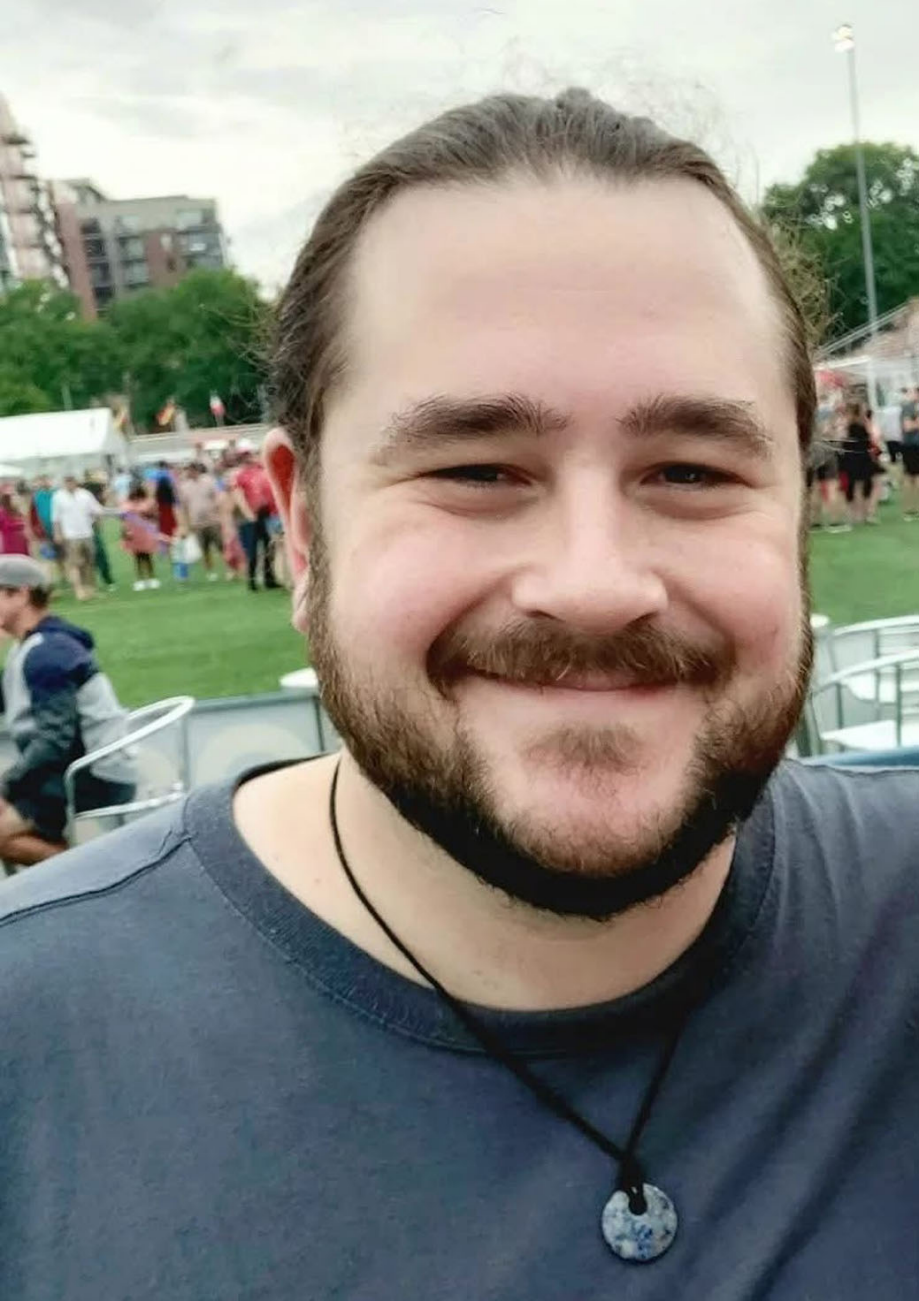


**Brandon Scharpf**

**Who or what inspired you to become a scientist?**

**B.S.:** For as long as I can remember, I have been fascinated by the complexity of life and the processes that led to what we observe in the natural world today. This childhood interest stemmed mainly from educational books, observing different animals at zoos and watching nature documentaries. Once in college, learning about the complexity and pathologies of different diseases, along with the many unanswered questions attached to them, sparked a desire to learn how to conduct research and contribute to the scientific community.

**H.R.:** As the daughter of two engineers, I planned on going to school for engineering all the way up to my senior year of high school. I grew up riding and training horses and had a horse develop an unpredictable neurological condition, which made him unsafe to ride. None of the veterinarians could figure out what was going wrong, so I ended up doing a lot of research looking for my own answers. This stemmed into me looking into options for a career as a veterinarian and researcher.

In this study, we identified cells specific to the prostate that produce collagen and that these cells come to the prostate in the blood.

**What is the main question or challenge in disease biology you are addressing in this paper? How did you go about investigating your question or challenge?**

**B.S.:** Lower urinary tract dysfunction (LUTD) is a disease nearly ubiquitous in ageing men. Current treatment options outside of surgery include drugs that target prostate size and smooth muscle dysfunction, but recent studies show that fibrosis of the prostate can contribute to the development of LUTD. For the development of new therapies, an understanding of the mechanisms and cell types mediating the disease is needed. We were able to identify a collagen-producing cell type, which colocalizes in areas of increased collagen density in LUTD patients, and a recruitment mechanism that collagen synthesis is dependent on. We did this by labelling and tracking different collagen-producing cell lineages in the *E. coli*-infected mouse prostate, identifying a phenotypically similar cell in histologically inflamed human prostate tissue and utilising *Ccr2* null mice to show that collagen synthesis is dependent on the CCL2–CCR2 signalling axis.

**H.R.:** When I joined the Vezina research lab, we had a general idea that prostatic fibrosis was correlated with worse urinary dysfunction in ageing men, but we didn't have the fibrosis fully characterized or know what was causing it. I did a few studies looking for collagen-producing cell types in aged dog prostates (who also develop ageing-related prostate enlargement and urinary dysfunction), but I was always met with the challenge that we were always looking at tissue where fibrosis had already occurred. In this project, we wanted to see what cells were producing collagen during active inflammatory events. Using lineage tracing, we could see where the cells came from. There was some support in prior literature that the cells responsible were of bone marrow origin. I added in the *Lyz2* lineage and *Cd2* lineage to specifically go after tracing myeloid and lymphoid lineages.


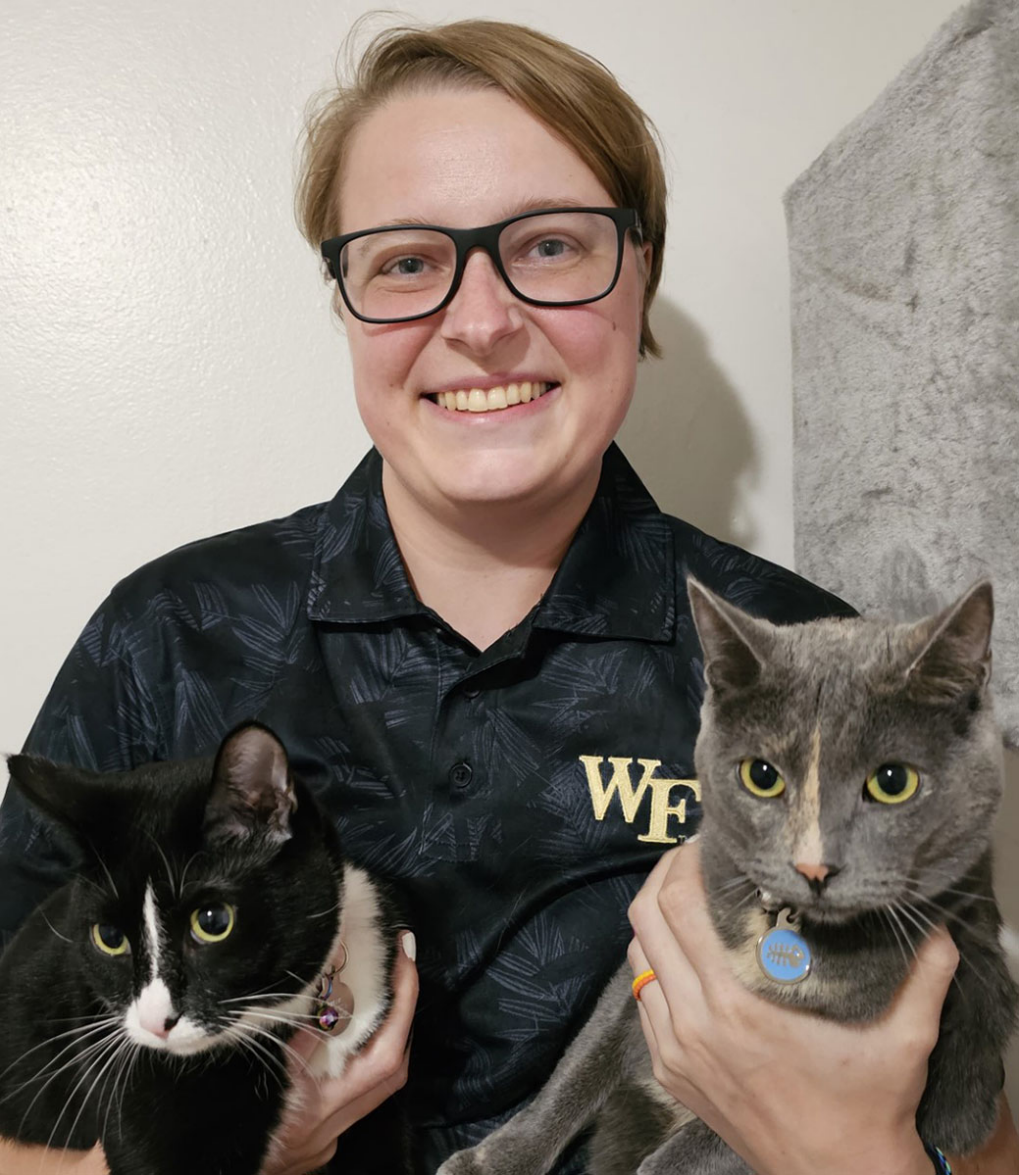


**Hannah Ruetten**

**How would you explain the main findings of your paper to non-scientific family and friends?**

**B.S.:** Collagen is a protein made by your body that provides structural support to tissues and is essential in the body's ability to repair tissues. The body's response to wound healing can be triggered by many different types of stimuli (e.g. infection, inflammation, toxin exposure, etc.). Normally, once the stimuli are dealt with and removed, tissue wound healing does its job and halts. If those stimuli never leave, then collagen is continuously deposited onto the site and can lead to fibrosis, or the interference of a organs normal function due to unchecked collagen accumulation. In our study, this process leads to the prostate stiffening and impedes the ability of urine to pass through the urethra. No current targeted treatments are available for fibrosis-induced LUTD, so we set out to identify key aspects of the mechanism at play. Understanding these mechanisms is necessary in order to develop targeted treatments.

**H.R.:** Collagen is one of the main building blocks of your body that gives everything shape and structure. When a build-up of collagen is causing disease, we can't give people a drug to just ‘remove collagen’ because then their entire body would be impacted – good collagen and bad collagen – and they would likely have lots of severe side effects. In this study, we identified cells specific to the prostate that produce collagen and that these cells come to the prostate in the blood. This finding is important because it gives us potential targets for drugs specific to ‘too much prostate collagen’ rather than all collagen in the body.

**What are the potential implications of these results for disease biology and the possible impact on patients?**

**B.S.:** These results can potentially be used as preclinical data for the development of drug treatments that target the mechanisms of fibrosis-induced LUTD. This can impact LUTD patients that cannot attribute their symptoms to benign prostate hyperplasia or smooth muscle dysfunction alone.

**H.R.:** Very broadly speaking, this study gives a framework for tracing cells influencing a disease process. In this application, we are looking for collagen-producing cells in the prostate during an acute inflammatory event, but the same approach could be used for tracing a number of different cell types in the body during different acute or chronic disease events. This study also identifies targets for preventative strategies to minimise collagen accumulation in the prostate.

**Figure:**
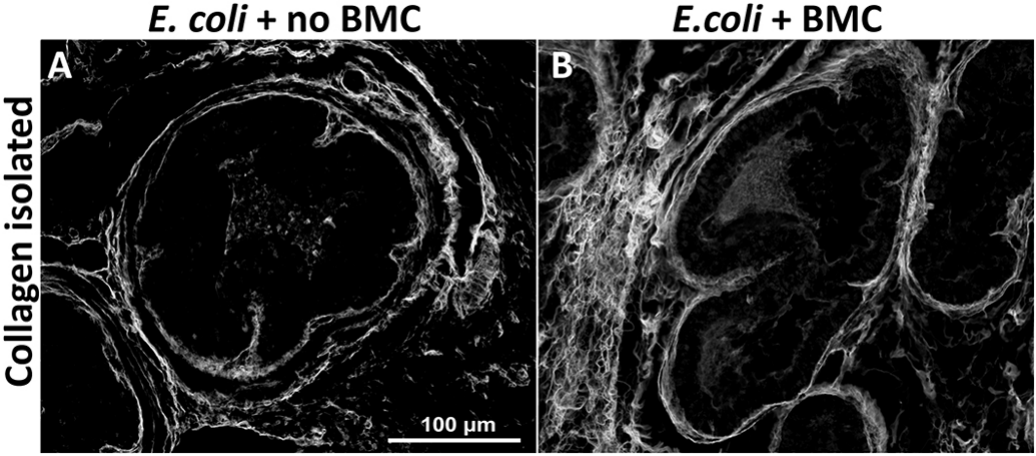
**Picrosirius Red-stained collagen fibres isolated in the dorsal prostate lobe of *Ccr2* null mice, infected with *E. coli* and with or without allotransplantation of Rosa-GFP donor bone marrow-derived cells (BMC).** (A) Without BMC. (B) With BMC.

**Why did you choose DMM for your paper?**

**B.S.:** DMM is a well-respected journal that focuses on mechanisms, diagnosis and therapy of human disease, and we believe our research fits DMM's mission well. We also believe that DMM's broad readership and open access will help connect our research to others interested in the field.

**H.R.:** DMM seems to have a strong focus on building a diverse scientific community filled with collaboration, and this is something that we also value.

A successful transition to independence really relies on support from your mentor/PI.

**Given your current role, what challenges do you face and what changes could improve the professional lives of other scientists in this role?**

**B.S.:** My current role is as a PhD student/research assistant. The challenges I face are typical to many PhD students, which include staying motivated, organized and accountable. I believe that improvements to organization will improve many scientists' productivity and motivation.

**H.R.:** As a postdoctoral fellow, I am in the in-between stage where I am working toward independence but still working for another lab. It seems like there are a lot of obstacles at this stage because it is hard to access time and monetary support to get preliminary data for your own ideas and independent career. A successful transition to independence really relies on support from your mentor/PI. It would be nice to see more small grants available for postdoctoral fellows with a small percent effort and some starting research funds to get strong preliminary data for an independent career.

**What's next for you?**

**B.S.:** We are working on a follow-up study focusing on the mechanistic side of things, specifically looking at the role of macrophages and how they mediate collagen synthesis in the cells we characterized in the current article. I am also working on finishing my graduate degree in the beginning of 2025.

**H.R.:** I am currently wrapping up some genitourinary-related regenerative medicine studies using cynomolgus macaque models and will be transitioning to a project looking at subtyping interstitial cystitis/bladder pain syndrome patients based on histopathology characteristics in bladder biopsies. I am looking forward to learning a bit more about cell culture and organoids, as well as working with human pathologists evaluating biopsy tissue and clinical data.

**Tell us something interesting about yourself that wouldn't be on your CV**

**B.S.:** My next biggest passion next to science is music and I have two cats, Oliver and Lucy, who are my biggest supporters.

**H.R.:** I grew up on a farm, so I try to bring a little farm life to every place I've lived. I currently have a flock of bantam chickens. When I was at University of Wisconsin-Madison I had a Silver Laced Polish hen, which I got right at the start of my PhD; she lives with my aunt now and will be 10 this year.
